# Association between vitamin D, vitamin D supplementation and benign paroxysmal positional vertigo: a systematic review and meta-analysis

**DOI:** 10.3389/fneur.2025.1560616

**Published:** 2025-04-16

**Authors:** Yanyan Li, Peng Gao, Rui Ding, Ying Xu, Zhicheng Wang, Xiaorui Pei, Lianhe Li

**Affiliations:** ^1^Department of Neurology Ward, Chaoyang Central Hospital of China Medical University, Chaoyang, China; ^2^Department of Reproductive Medicine, Chaoyang Central Hospital of China Medical University, Chaoyang, China; ^3^Department of Laboratory of Molecular Biology, Chaoyang Central Hospital of China Medical University, Chaoyang, China; ^4^Department of Head and Neck Surgery Ward, Chaoyang Central Hospital of China Medical University, Chaoyang, China

**Keywords:** BPPV, 25-hydroxy vitamin D, recurrence, vitamin D supplementation, canalolithiasis, cupulolithiasis

## Abstract

**Background:**

Benign paroxysmal positional vertigo (BPPV) is one of the most prevalent peripheral vestibular dysfunctions encountered in clinical practice, including dizziness and vertigo, which has a significant impact on people’s everyday lives and affects their quality of life in many ways. Researches indicate that individuals with recurrent benign paroxysmal positional vertigo (BPPV) may exhibit vitamin D insufficiency, and certain studies suggest that correcting severe vitamin D deficiency might effectively reduce BPPV recurrence; nevertheless, the findings have been inconsistent. As a result, we conducted the current Meta-analysis to investigate potential associations of vitamin D levels with the occurrence and recurrence of BPPV. In the meantime, the current study was done to evaluate the impact of vitamin D supplementation on the prevention of benign paroxysmal positional vertigo recurrence.

**Methods:**

Electronic databases (PubMed, EMBASE, SCOPUS and the Cochrane Library) were identified to search for relevant studies about (vitamin D or vitamin D supplementation) and (Benign paroxysmal positional vertigo incidence or recurrence) from inception to Dec 22, 2024. 60 studies with a total of 16,368 participants were included into this meta-analysis.

**Results:**

(1) The aggregated weighted mean difference (WMD) demonstrated that there was a significant reduction in vitamin D level in the BPPV cohort (WMD = −2.84; 95% CI −4.53 to −1.15) relative to the control cohort. Likewise, Recurrent BPPV groups had significantly lower levels of vitamin D compared to non-recurrent groups (WMD = −5.01; 95% CI −6.94 to −3.08). When the cupulolithiasis BPPV groups were compared to the canalolithiasis BPPV groups, the vitamin D level was lower in the cupulolithiasis groups (WMD = 5.09; 95% CI 2.05 to 8.12); (2) In this meta-analysis, the multivariable-adjusted relative risk (RR) indicated that increased vitamin D was inversely related to BPPV incidence (RR = 1.36; 95% CI 1.31, 1.41), but not significantly related to the recurrence (RR = 0.95, 95% CI 0.91, 0.99); (3) Vitamin D supplementation group had a lower recurrence rate than the control group which did not accepted vitamin D supplementation (RR =0.45, 95% CI = 0.36–0.55).

**Conclusion:**

The serum level of vitamin D is lower in patients with BPPV, especially recurrent BPPV, than in controls. There was a negative correlation between occurrence rate of BPPV episodes and vitamin D deficiency, which means that vitamin D deficiency may have a role in occurrence of BPPV. The present study indicates that vitamin D supplementation can significantly lower recurrence in benign paroxysmal positional vertigo. The level of vitamin D was lower in canalolithiasis than in cupulolithiasis BPPV groups.

## Introduction

Benign paroxysmal positional vertigo (BPPV) is the predominant kind of peripheral vestibular dysfunction, produced by the displacement of otoconia from the otolith organs into the semicircular canals, resulting in clinical manifestations such as vertigo, vomiting, and dizziness (1). The diagnostic criteria for benign BPPV were formulated by the Committee for the Classification of Vestibular Disorders of the Bárány Society (2), which is identified by characteristic positioning nystagmus consistent with different semicircular canal otolith and clinical symptoms (3). In approximately 50% of BPPV patients, the condition arises from idiopathic origins (4), often linked to age-related degenerative processes. Among secondary factors, traumatic head injuries account for roughly 17% of BPPV cases (5), other reasons include serum vitamin D deficiency, osteoporosis, vascular risk factors and so on (6). In an episode of benign paroxysmal positional vertigo (BPPV), over 86% of patients have severe vertigo, which forces them to interrupt their work immediately, thus having a negative impact on their daily lives (7). BPPV typically recurs, with a recurrence rate of 15–56% during a 1–10 year period (6). This not only results in increasing medical expenses for individuals and their families but also imposes an additional burden on society in terms of healthcare resources utilization. Therefore, it is crucial to ascertain the variables that contribute to the onset and recurrence of benign paroxysmal positional vertigo (BPPV).

The primary components of otoconia are CaCO3 and glycoprotein crystals, which are connected to hair cells via protein fibers. The otoconia crystals are formed by the vestibular organ’s active calcium metabolism activities (8). Otoconia crystals possess a core nucleus predominantly made of organic glycoproteins with little calcium (Ca) content. The crystals are encircled by inorganic outer zones composed mostly of CaCO3, exhibiting elevated calcium concentrations (9). Vitamin D, a lipid-soluble prehormone, is essential for regulating calcium and phosphorus levels. Vitamin D can be gained by dietary intake, consumption of vitamin D-fortified foods, and skin exposure to UVB radiation (10). Adequate amounts of vitamin D are required for optimal calcium metabolism, which influences the function of the otoliths (11). Furthermore, vitamin D receptors are located in several cells of the inner ear, including those associated with the vestibular system. Insufficient vitamin D is considered to disrupt normal signaling pathways and cellular processes in these receptors, hence leading to vestibular dysfunction (12). According to a prior epidemiological investigation, the risk of BPPV recurrence rose from 15% in the first year following CRP therapy to 50% over the course of three years (6). Vitamin D affects calcium and bone metabolism, which has been linked to BPPV development.

Numerous investigations have demonstrated that low blood levels of 25-hydroxyvitamin D (25(OH)D) are associated with both the incidence and recurrence of BPPV (13–15), vitamin D insufficiency has been extensively studied as a risk factor for the development and recurrence of BPPV. However, the results are inconclusive. Talaat et al. (13) found that low levels of 25(OH)D were linked to the onset of BPPV and the recurrence of BPPV. Ding et al. (14) demonstrated 25(OH)D insufficiency was connected with BPPV incidence and recurrence, as well as negatively associated with the severity of BPPV. Some researchers have confirmed that vitamin D is not related to the pathogenesis of BPPV and its recurrence (16–18). The researchers reached contradictory conclusions.

Patients suffering from the various kinds of BPPV can be effectively treated with a suitable single canalith repositioning therapy in up to 85% of instances (19). Although the first therapy is effective, the 1-year recurrence rate of BPPV is reported to be between 13.7 and 48.0% (20). Several studies recently reported a greater frequency of vitamin D deficiency/insufficiency in individuals with BPPV than in controls (6, 12–26). Given these findings, the question of vitamin D supplementation arises. In reality, vitamin D treatment may reduce the recurrent episodes of BPPV, and curiously, a full remission was achieved in a large number of individuals after trials of vitamin D supplementation (27–30). Supplementation is intended to address the deficit and maybe improve the condition of BPPV sufferers. Some studies have also suggested that vitamin D intake can reduce recurrences of BPPV (13), but Rhim et al. (31)cannot get the same conclusion. So whether patients with benign paroxysmal positional vertigo (BPPV) can reduce the risk of BPPV by supplementing vitamin D remains controversial.

The primary purposes of this meta-analysis are to identify the relationship among vitamin D and BPPV occurrence, vitamin D and BPPV incidence, also summarize the evidence for supplementation of vitamin D on BPPV recurrences.

## Materials

### Search strategy and study selection

A thorough literature search was carried out from the beginning to Dec 22th, 2024, using MEDLINE, PubMed, the Cochrane Library, Web of Science, and the EMBASE databases, the following search phrases and keywords were linked by “and” or “or”: (Benign Paroxysmal positional vertigo or otolithiasis or BPPV) and (vitamin D or 25(OH) vitamin D or 25-hydroxylvitamin D or vitamin D supplementation). The study was done using the Preferred Reporting Item for Systematic Reviews and Meta-Analysis (PRISMA) standard (32) and was preregistered in PROSPERO (CRD420251002488, see Supplementary file-PROSPERO).

## Methods

### Data extraction and study quality

#### Inclusion criteria

The search methodology was confined to publicly accessible data and articles in the English language. Publications were selected according to the following criteria: (1) The investigations were comparative studies carried out retrospectively or prospectively on adult patients. (2) examined the relationship between vitamin D, vitamin D supplementation and Benign Paroxysmal positional vertigo; (3) Vitamin D was measured using a recognized technique and represented as, or converted to, one international unit (ng/ml), providing relative risk (RR), odds ratio (OR), or weighted mean difference (WMD) with a 95% confidence interval. (4) Diagnosis of BPPV: The diagnostic criteria for benign BPPV were formulated by the Committee for the Classification of Vestibular Disorders of the Bárány Society (2). BPPV diagnosis was based on a characteristic history and observation of typical nystagmus during the Dix-Hallpike manoeuvre, supine roll, and cephalic hyperextension tests. For posterior semicircular canal BPPV, the Dix-Hallpike test was positive if nystagmus was recorded with appropriate positioning, latency, duration, and fatigability. Lateral semicircular canal BPPV (LC-BPPV) was diagnosed by positional nystagmus changing in the horizontal direction concurrent with vertigo triggered by the supine roll test. According to the direction of the nystagmus as horizontal geotropic and apogeotropic nystagmus, the LC-BPPV was classified as canalolithiasis or cupulolithiasis, respectively. A “recurrence” was defined as a new BPPV episode occurring at least 2 weeks after verifying the complete resolution of the previous one.

#### Exclusion criteria

Two reviewers (Peng Gao and Rui Ding) independently retrieved data from pertinent articles utilizing data extraction forms. Any discrepancies between the two reviewers were resolved through discussion with another author (YY Li). We excluded: (1) duplicate or irrelevant papers; (2) reviews, letters, or case reports, commentaries; (3) non-original research; (4) non-human subjects.

#### Data extraction

The impact estimates corrected for the maximum number of confounding factors, together with their 95% confidence intervals (CIs), for the comparison between the highest and lowest vitamin D levels, were obtained. Additionally, the vitamin D levels (mean ± standard deviation [SD]) were obtained for both BPPV and control patients. Conversely, the unit of circulating selenium was standardized to “ng/mL” throughout all trials.

#### Quality assessment

The two reviewers (Peng Gao and Rui Ding) independently assessed the quality and risk of bias of the included studies using the Newcastle-Ottawa score (NOS) (33); All disagreements between the two reviewers were fully discussed, and furthermore a third reviewer (YY Li) was consulted for unresolved discrepancies to reach a consensus. Researches that received scores of 0–4 and 5–9 were classified as high-quality and low-quality, respectively.

### Statistical analysis

The meta-analysis was conducted using STATA version 17.0 and Review Manager software version 5.3. For continuous data, mean ± standard deviation is displayed, if the included studies provided the data using median and quartile values, we estimated the mean and standard deviation 17 using the Wan et al. Approach (34) Statistical significance was defined as *p*-values<0.05, and 95% confidence intervals were supplied. The weighted mean difference (WMD) was used as the metameter and the standard deviation (SD) was considered in evaluating the precision and significance of that point of estimate. Whereas heterogeneity across studies was evaluated using Cochrane-based Q and I (2) tests. Data followed by *p* < 0.05 or I (2) > 50% were considered to denote statistically significant heterogeneity, and were subjected to a randomized-effects model. Otherwise, a fixed-effects model was used. A funnel plot was used to determine publication bias.

## Results

### Study characteristics

#### Literature search and study characteristics

**Figure 1:**
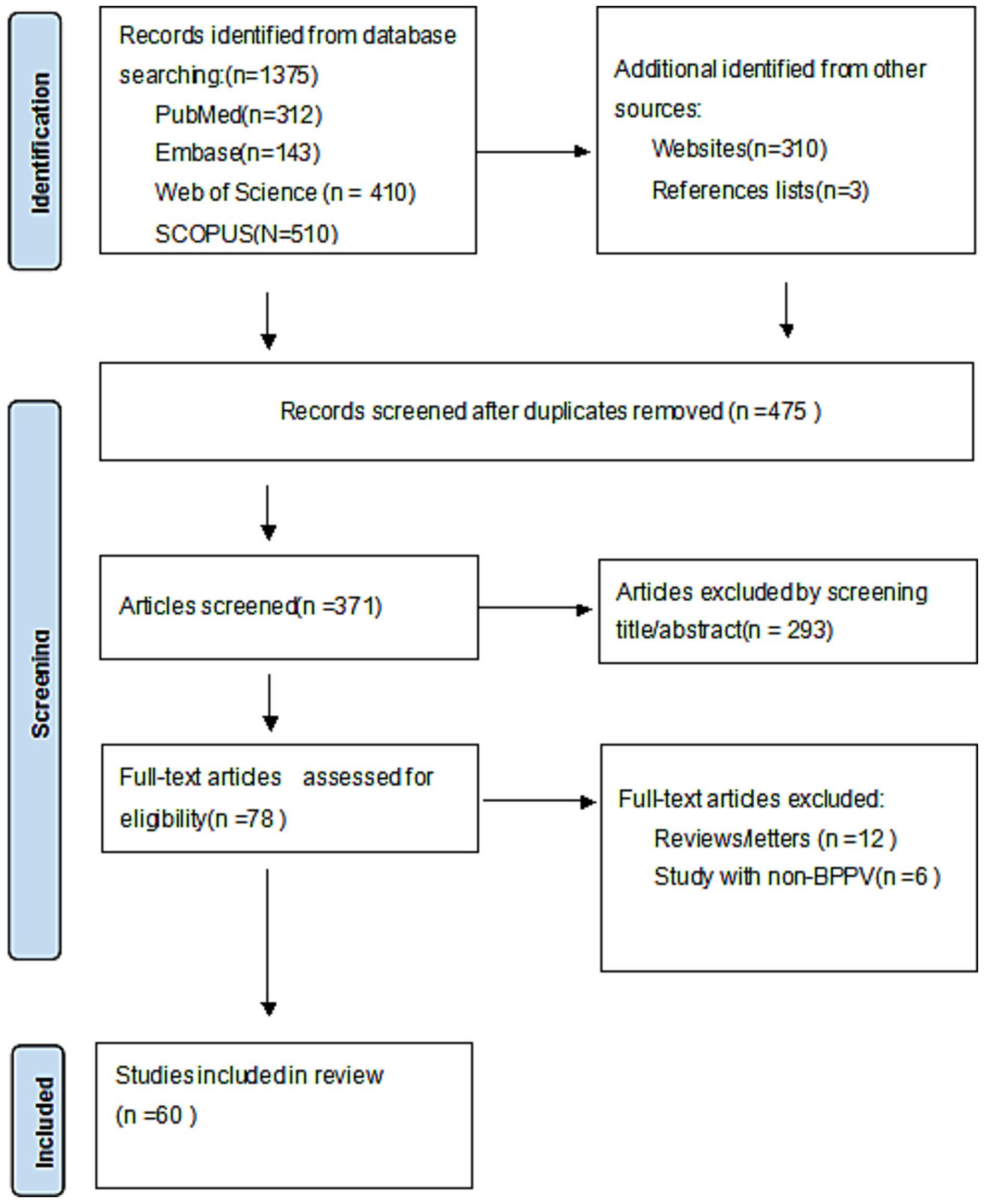
Preferred reporting item for systematic reviews and meta-analysis (PRISMA) guideline.

A total of 1,375 citations were located, and only the titles and abstracts passed muster. Full text of potentially relevant papers was read. Tables 1, 2 lists the features of the included studies, and Figure 1 displays the flow chart of the literature search. After removing the duplicates, 475 items were still present, 293 articles were excluded by screening the titles and abstracts. 12 reviews and 6 non-BPPV studies were removed. Eventually, the remaining sixty full-text papers were assessed based on the qualifying requirements (Figure 1).

**Table 1:** Characteristics of the included studies.

Study and year	Country	Study type	N total	Case (umol/l)	Control (umol/l)	Vitamin D in recurrent	Vitamin D in non-recurrent	OR (incidence/recurrence)	Quality-NOS
Zhang, 2020 (41)	China	Case–control	206	18.8 + −2.5	24 + −2.2	NA	NA	3.045 (1.467,4.638)	9
Yang, 2017 (3)	Korea	Case–control	260	18.2 + −10.3	20 + −8.1	NA	NA	NA	8
Yadav, 2021 (21)	India	Case–control	46	26.7 + −16.09	15.74 + −5.78	NA	NA	0.28 (0.1,0.85)	7
Wu, 2018 (22)	China	Case–control	152	20.99 + −6.76	23.17 + −6.49	NA	NA	3.8(1.25,11.73)	9
Wang, 2021 (23)	China	Case–control	36	20.7 + −1.95	30.59 + −2.75	NA	NA	0.57 (0.13,1.03)	9
Wang, 2019 (26)	China	Case–control	183	17.15 + −2.03	23.85 + −3.13	NA	NA	2.0 (0.94,3.33)	6
Thomas, 2022 (50)	India	Case–control	98	21.26 + −0.57	17.59 + −0.06	NA	NA	NA	7
Talaat, 2014 (13)	Egypt	Case–control	180	16.04 + −10.26	19.53 + −8.45	11.93 + −7.57	16.04 + −10.26	4.54(1.41,14.58)	7
Song, 2020 (36)	China	Case–control	320	16.89 + −7.45	15.95 + −8.06	NA	NA	1.57 (1.21,2.02)	8
Sarsitthithum, 2023 (35)	Thailand	Case–control	137	21.5 + −5.3	26.3 + −6.8	NA	NA	1.51(1.32,1.72)	8
Saeed Al-Rawi, 2024 (43)	Ramadi	Case–control	160	15.46 + −6.14	23.6 + −12.58	12.62 + −4.1	18.3 + −6.61	0.74 (0.50,1.10)	8
Resuli, 2022 (6)	İstanbul	Case–control	358	18.8 + −6.1	30.74 + −8.53	NA	NA	NA	8
Ren, 2023 (20)	China	Case–control	364	17.17 + −2.29	19.26 + −2.39	NA	NA	1.643 (1.058,2.550)	7
Pecci, 2022 (15)	Italy	Case–control	50	20.18 + −9.26	23.73 + −8.42	NA	NA	0.968 (0.914,1.026)	8
Parham, 2013 (25)	USA	Case–control	29	10.32 + −0.92	9.98 + −0.96	NA	NA	0.50 (0.31, 0.80)	8
Melis, 2020 (40)	Italy	Case–control	120	26.1 + −11.66	46.02 + −12.56	NA	NA	0.86 (0.76, 0.96)	8
Lee, 2017 (37)	Korea	Case–control	184	34.2 + −14.3	30.3 + −18.6	NA	NA	2 (1.9,2.1)	7
Kim, 2020 (65)	Korea	Case–control	78	17.54 + −8.93	15.61 + −9.76	NA	NA	3.2(0.8,12.1)	7
Karataş, 2017 (16)	Turkey	Case–control	156	23 + −14.4	17 + −12.3	NA	NA	1.66 (0.87,3.17)	9
Kahraman, 2016 (39)	Turkey	Cohort	74	9.73 + −8.77	19.08 + −5.92	NA	NA	NA	7
Jeong, 2013 (24)	Korea	Case–control	292	14.4 + −8.4	19.1 + −6.8	NA	NA	3.8(1.51,9.38)	8
Isik, 2017 (9)	Turkey	Case–control	127	9.51 + −5.49	11.02 + −9.62	NA	NA	0.71(0.56,0.90)	6
Inan, 2021 (38)	Turkey	Case–control	104	15.3 + −9.8	20.2 + −14.3	NA	NA	0.68 (0.56,0.83)	8
Hualan Yang, 2017 (12)	China	Case–control	102	23.13 + −6.11	26.85 + −5.92	NA	NA	NA	8
Han, 2018 (19)	China	Case–control	165	19.1 + −5.2	22.5 + −5.8	NA	NA	2.1 (1.12, 3.95)	8
Goldschagg, 2021 (18)	Germany	Case–control	379	23.4 + −9.4	24.9 + −10.1	NA	NA	0.62 (0.42, 0.92)	7
Ding, 2019 (14)	China	Case–control	522	18.22 + −2.16	21.85 + −1.6	NA	NA	2.15(1.30,4.32)	8
Cobb, 2022 (48)	USA	Case–control	6,135	31.4 + −16.5	26 + −11.2	29 + −12	37.6 + −18.3	0.47 (0.32, 0.63)	8
Cheng, 2021 (1)	China	Case–control	640	23.2 + −4.09	25.8 + −3.43	NA	NA	0.88 (0.83,0.94)	8
Chauhan, 2023 (46)	India	Case–control	88	27.9 + −15.89	39.05 + −21.15	NA	NA	0.71(0.53,0.93)	9
Bi, 2021 (47)	China	Case–control	52	14.64 + −6.94	19.56 + −6.55	NA	NA	1.073 (0.964,1.194)	8
Bener, 2024 (55)	Turkey	Case–control	833	19.04 + −8.37	21.19 + −9	NA	NA	1.32(0.59,2.96)	9
Bazoni, 2019 (44)	Brazil	Case–control	109	27.8 + −10.1	23.8 + −11.28	NA	NA	0.57(0.,22,1.48)	8
Büki, 2013 (28)	Austria	Cohort	18	NA	NA	13.41 + −1.9	28.77 + −11.13	NA	8
Chen, 2017 (20)	China	Case–control	249	NA	NA	18.1 + −6.6	19 + −7.6	NA	8
Chu, 2024 (42)	Singapore	Case–control	149	NA	NA	17.4 + −5.25	21.4 + −5.02	0.83(0.76,0.90)	8
Ding, 2019 (14)	China	Case–control	174	NA	NA	11.85 + −3.29	18.77 + −2.02	5.16 (1.00,34.12)	9
Libonati, 2020 (56)	Italy	Rct	109	NA	NA	18.2 + −6.6	36.9 + −5.7	NA	9
Lin 2024 (49)	China	Case–control	138	NA	NA	13.37 + −4.62	18.15 + −6.29	NA	8
Maslovara, 2018 (17)	Croatia	Cohort	31	NA	NA	22.88 + −13.77	19.11 + −6.27	NA	8
Melis, 2020 (40)	Italy	Cohort	73	NA	NA	19.53 + −15.33	25.85 + −14.1	NA	8
Rhim, 2016 (29)	Korea	Cohort	232	NA	NA	13.64 + −6.97	16.63 + −7.4	NA	7
Rhim, 2020 (31)	Korea	Case–control	332	NA	NA	14.1 + −13.5	14.9 + −13.9	0.976 (0.934, 1.020)	8
Shin, 2022 (45)	Republic of Korea	Cohort	50	NA	NA	12.9 + −8	19.2 + −8.2	NA	8
Yang, 2017 (3)	Korea	Case–control	126	NA	NA	19.3 + −11.1	17.2 + −9.4	NA	8
Zhang, 2018 (65)	China	Case–control	78	NA	NA	17.15 + −6.04	18.45 + −4.28	NA	7
Zhang, 2020 (41)	China	Case–control	156	NA	NA	16.7 + −2.1	19.3 + −2.3	NA	9

NA, not available; NOS, Newcastle-Ottawa Scale; N, number; OR, odds ratio.

**Table 2:** Effects of vitamin D supplementation and BPPV recurrence observed in this review.

Study and year	case/control	intervention	Follow up (months)	vitamin D before treatment	vitamin D after treatment	P for recurrence difference
Büki, 2013 (28)	4/14	8,000 IU cholecalciferol daily for two weeks, and daily 4,000 IU cholecalciferol for the next two weeks, then a weekly dose of 8,000 IU	8	14	27	*p* < 0.02
Carneiro de Sousa, 2014 (29)	71/57	5,000 IU of cholecalciferol vitamin D daily for 12 months	12	14	30.6	*p* = 0.001
Califano, 2014 (30)	68/68	10,000 IU to 50,000 IU of cholecalciferol Vitamin D weekly for 12 months	12	18.2 + −10.43	NA	*p* < 0.001
Sheikhzadeh, 2016 (58)	27/27	5,000 IU of cholecalciferol vitamin D daily for 2 months	6	11.41 ± 1.9	34.2 ± 3.3	P = 0.001
Talaat, 2016 (13)	28/65	50,000 IU oral vitamin D3, 3 times/week, for 1 month, followed by 50,000 IU oral vitamin D3, once every 2 weeks plus Calcium citrate 600 mg tablets twice daily	18	6.7 + −2	28.3 + −5	*P* < 0.001
Rhim, 2020 (31)	40/45	Intramuscular injection 200,000 IU, three to four injections in the first year, The injection solution contains 200,000 IU (5 mg) of cholecalciferol	24	6.06 + −3.5	31.1 + −6.8	*p* = 0.883
Jeong, 2020 (57)	445/512	Vitamin D 400 IU and 500 mg of calcium carbonate twice a day for 1 year.	12	13.3 + −3.9	24.2 + −8.4	*p* = 0.005
Elmoursy, 2021 (53)	20/20	NA	6–12	NA	NA	*p* = 0.047
Abdelmaksoud, 2021 (54)	20/20	Cholecalciferol 8,000 IU daily for 2 weeks followed by 4,000 IU daily for 2 weeks then 8,000 IU single dose weekly for 3 months	6	**12.4 ± 2**	26.3 ± 4.1	P = 0.000
Pecci, 2022 (15)	26/24	50.000 IU once a week for 2 weeks, then 25.000 IU once a week for 2 weeks, then 7.000 IU once a week for 2 months	4–8	20.18	28.1	*p* = 0.0003
Sharma, 2022 (52)	20/20	Consisted of daily dose of 5,000 IU cholecalciferol for one month, then 5,000 IU cholecalciferol twice weekly for one month, then a weekly dose of 5,000 IU was given thereafter.	6	12.3	27.2	P = 0.001
Sánchez, 2022 (27)	17/18	Colecalcifero 1,600 U once a week during 8–10 weeks	6–13	18.5 + −6.8	26.2 + −4.9	*p* = 0.017
Kong, 2024 (51)	20/18	7,000 IU of vitamin D weekly for a year	6–12	12.0 ± 4.8	28.2 ± 6.5	*p* = 0.003

Subgroup of vitamin D supplementation and BPPV recurrence.

NA, not available.

Consequently, a total of 60 studies involving BPPV, vitamin D, vitamin D supplementation and the recurrence of BPPV, were examined qualitatively, and then a meta-analysis was conducted. (Table 1) 0.47 studies about vitamin D and incidence, recurrence of BPPV with a total of 14,654 patients, 13 studies about, vitamin D supplementation and the recurrence of BPPV, totaling 1714 participants, were analyzed qualitatively and meta-analyzed, details of the included studies are shown in Table 2. Seven to nine was the range of quality scores. Every record that was included was thought to be of high caliber.

#### The aggregated weighted mean difference difference (WMD) of the vitamin D level between BPPV and controls, non-recurrence BPPV and recurrence BPPV

**Figure 2:**
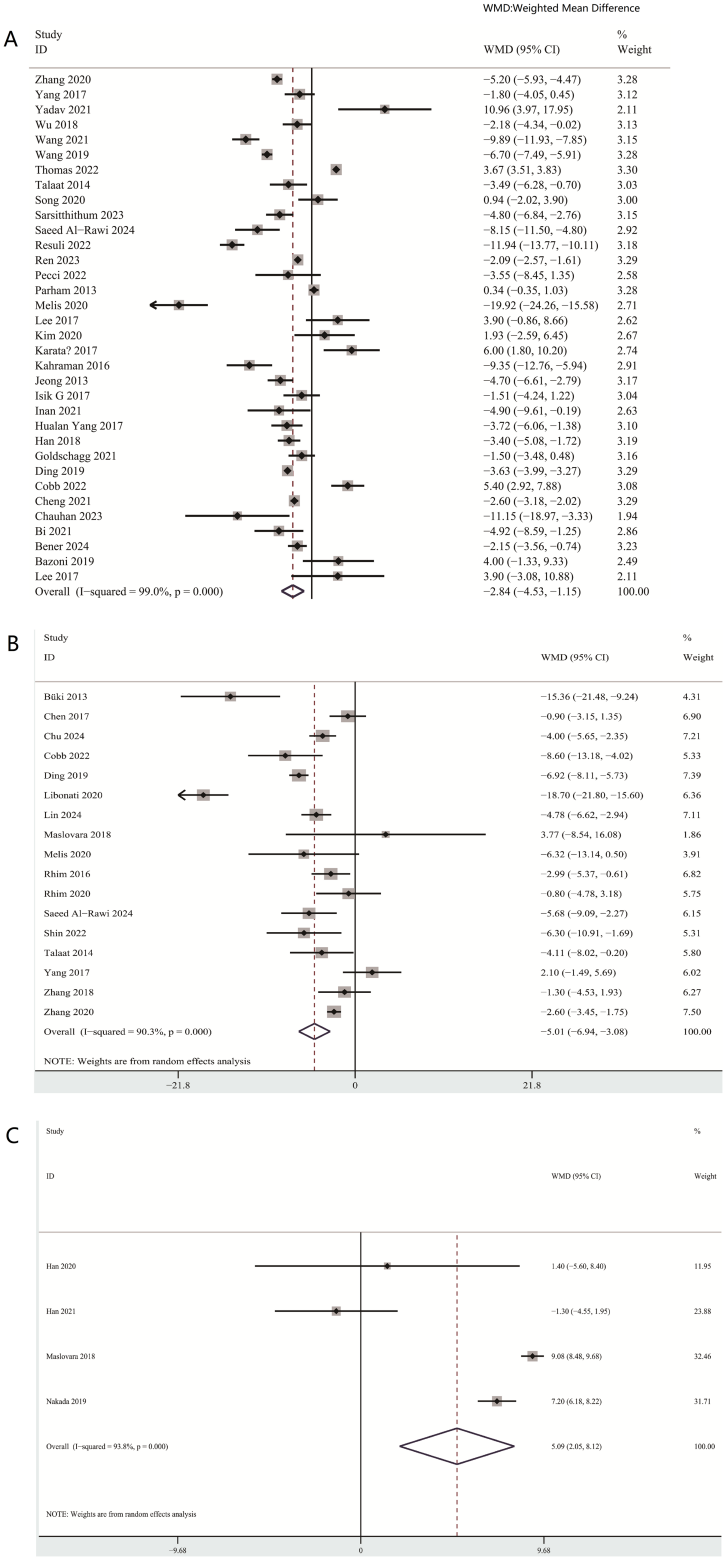
Vitamin D levels between BPPV and controls, non-recurrence BPPV, and recurrence BPPV **(A)** Vitamin D level between BPPV and controls **(B)** Vitamin D level between non-recurrence BPPV and recurrence BPPV **(C)** Vitamin D level between cupulolithiasis and canalolithiasis BPPV groups.

The aggregated weighted mean difference (WMD) demonstrated that there was a significant reduction in vitamin D level in the BPPV cohort (WMD = −2.84; 95% CI −4.53 to −1.15) (Figure 2A) relative to the control cohort. Likewise, when the recurrent BPPV groups were compared with the non-recurrent BPPV groups, the statistical analysis showed significantly lower level of vitamin D among the recurrence BPPV groups (WMD = −5.01; 95% CI −6.94 to −3.08) (Figure 2B).

#### The aggregated mean difference (WMD) of the vitamin D level between cupulolithiasis and canalolithiasis BPPV groups

When the cupulolithiasis BPPV groups were compared with the canalolithiasis BPPV groups, the vitamin D level showed lower level of vitamin D in the cupulolithiasis groups (WMD = 5.09; 95% CI 2.05 to 8.12) (Figure 2C).

#### Multivariable-adjusted RR of BPPV for the highest compared with lowest circulating vitamin D level category

**Figure 3:**
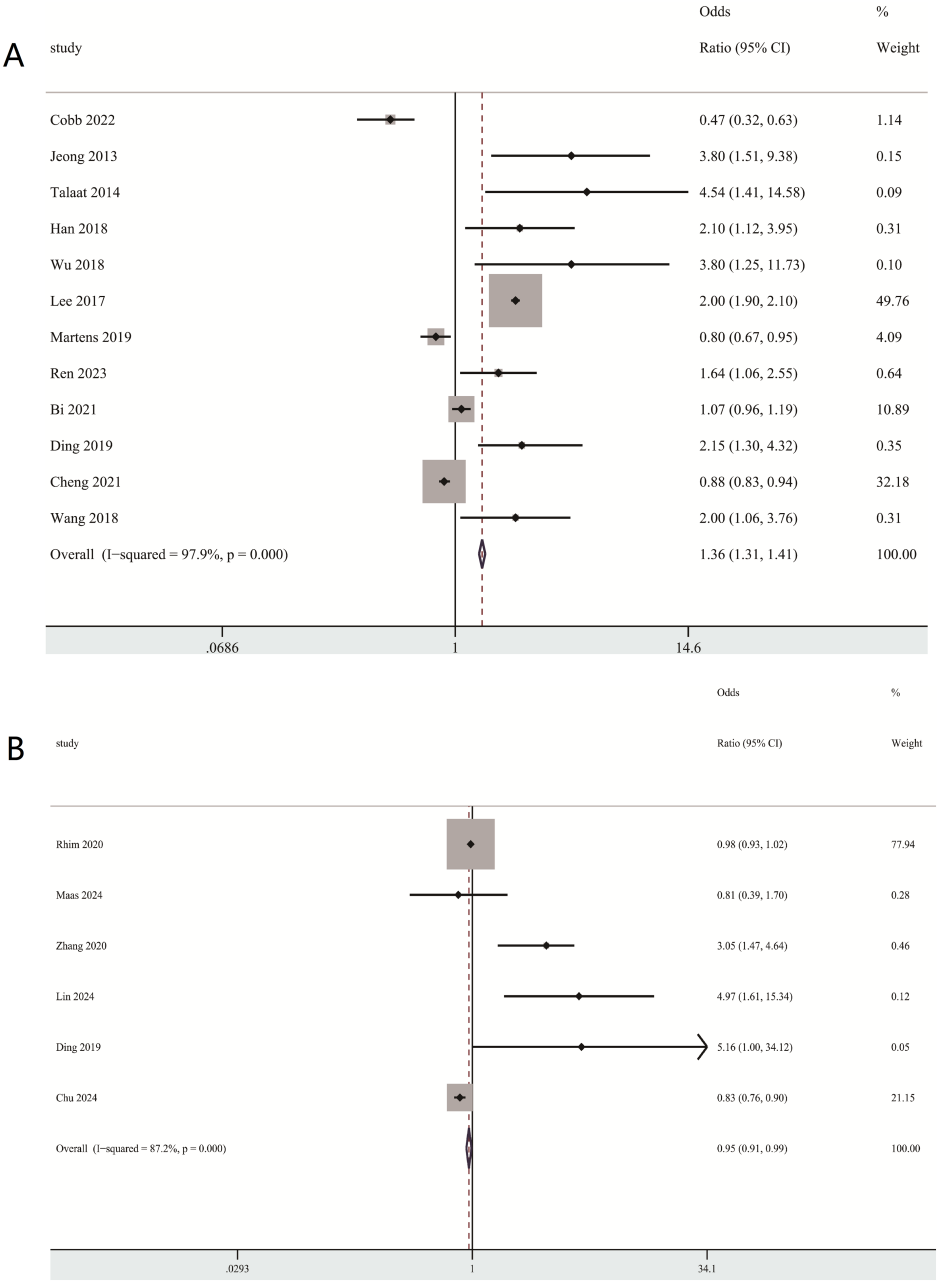
Association between vitamin D levels and BPPV **(A)** Association between vitamin D levels and BPPV risk **(B)** Association between vitamin D levels and recurrence of BPPV risk.

In this meta-analysis, the multivariable-adjusted relative risk (RR) indicated that increased circulating vitamin D was inversely related to BPPV incidence (RR = 1.36 95% CI 1.31, 1.41) (Figure 3A), but not significantly related to the recurrence (RR = 0.95, 95% CI 0.91, 0.99) (Figure 3B).

#### The connection between vitamin D supplementation and BPPV recurrence

**Figure 4:**
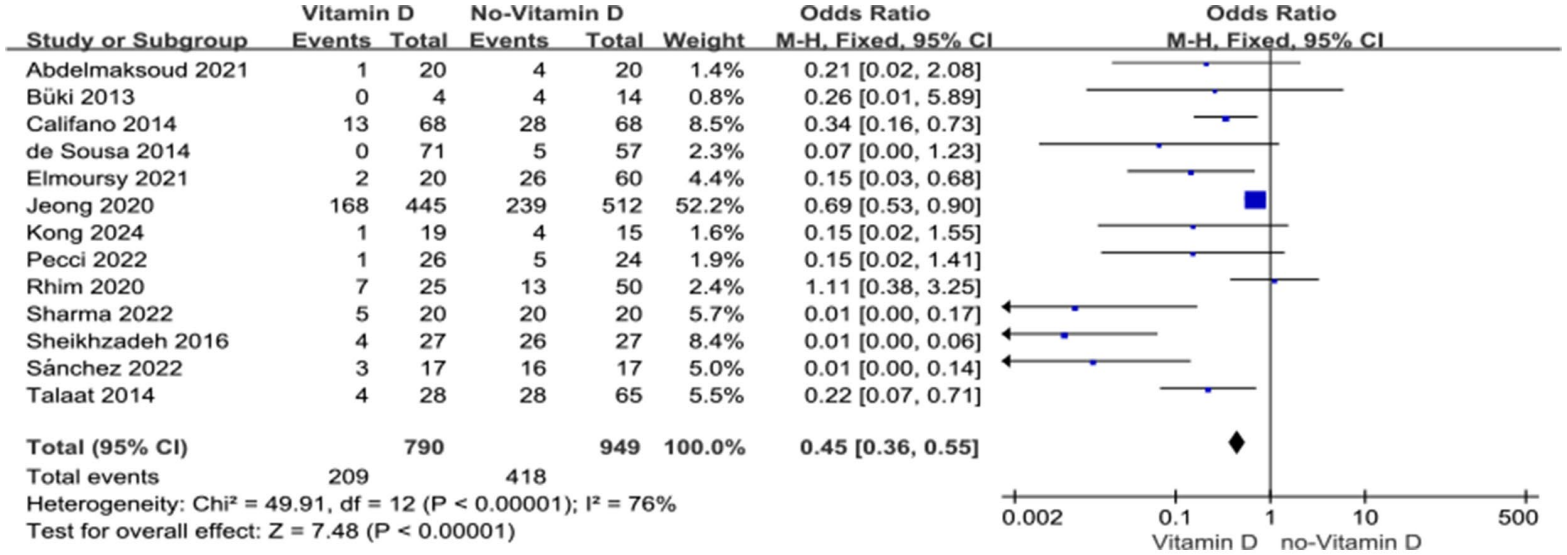
Vitamin D supplementation and BPPV recurrence.

Vitamin D supplementation group had a lower recurrence rate than the control group who did not accepted vitamin D supplementation (RR =0.45, 95% CI = 0.36–0.55) (Figure 4).

#### Publication bias

**Figure 5:**
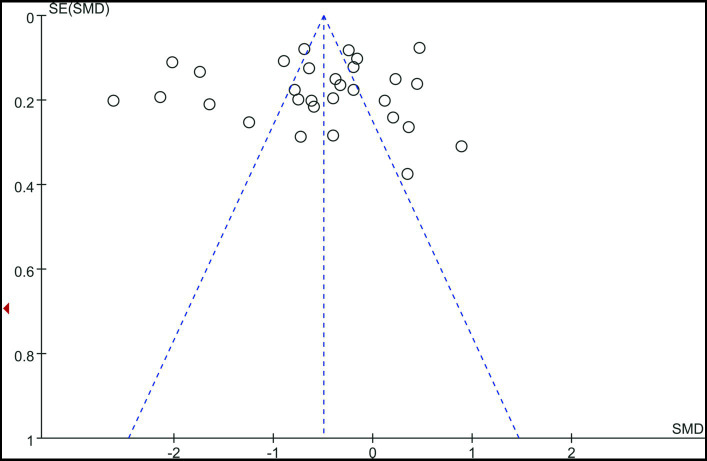
Funnel plot.

The funnel plot was basically symmetrical, and there was no evidence of publication bias (Figure 5).

## Discussion

Our meta-analysis indicates that blood vitamin D levels are lower in BPPV patients, particularly those with recurrent BPPV, than in controls. There was a negative association between the frequency of BPPV episodes and vitamin D deficit, indicating that vitamin D deficiency may play a role in the development of BPPV. Likewise, our current meta-analysis demonstrates that lower vitamin D levels are significantly related with BPPV incidence rather than recurrence. The current investigation found that vitamin D supplementation lowers BPPV recurrence in patients with vitamin D insufficiency. At the same time, we found that vitamin D levels were lower in cupulolithiasis than in canalolithiasis groups.

In vitamin D receptor null mice, otoconia exhibited degenerative characteristics such as fissures, fusion, and tiny particles. These data revealed a substantial link between BPPV and vitamin D insufficiency (18). Many studies (34–39) focus on the vitamin D level and BPPV populations, which included incidence and recurrence of BPPV, but got inconsistent conclusions. Melis et al. (40) suggested a relationship between vitamin D deficiency and BPPV onset. Jeong et al. (24) also shows lower level 25-OH vitamin D concentrations in patients with BPPV when compared with control group. Zhang et al. (41) conducted a prospective study found that vitamin D level is lower in BPPV groups. In Chu et al. (42), Saeed Al-Rawi et al. (43), and Ding (14) study, lower levels of vitamin D was associated with the recurrence of BPPV. On the contrary, Işık et al. (9) found no correlation between vitamin D levels and BPPV occurrence or recurrence. Bazoni (44) et al. and Kim et al. found that vitamin D levels are elevated in patients with BPPV. Shin et al. (45)and other researchers (46–48) found that lower serum vitamin D level is a risk factor for BPPV recurrence. Likewise, Lin et al. (49) indicated that 25(OH)-D levels were markedly decreased in recurrent BPPV group compared to a non-recurrent BPPV subgroup. Nevertheless, a Croatian investigation indicated that a diminished blood level of vitamin D3 did not elevate the probability of BPPV recurrence (17). Another investigation conducted in India revealed that there is no correlation between calcium and vitamin D levels and BPPV (50). Our meta-analysis were consistent with Talaat et al. (13) study, which showed that the BPPV population had a lower blood vitamin D levels than the controls. Meanwhile, our systematic review confirmed that, compared with non-recurrent BPPV population, patients with recurrent BPPV had lower vitamin D levels, which proves that BPPV and its recurrence are associated with lower serum vitamin D levels. Unfortunately, our systematic review cannot prove a causal relationship between the occurrence and recurrence of BPPV and vitamin D levels.

Although a causal relationship cannot be showed by a retrospective investigation, if lower vitamin D levels are associated with BPPV in this subgroup, it is fair to believe that supplementation may have a role in avoiding BPPV development. For example, Kong et al. (51) and other researchers (52–55) established a link between vitamin D supplementation and a reduction in BPPV recurrence. They revealed that daily supplementation with oral vitamin D and calcium carbonate over the year of therapy significantly reduced the recurrence of BPPV in people with lower blood vitamin D levels. Many studies (42, 55) established a negative correlation between the recurrence rate of BPPV and vitamin D deficit, suggested that vitamin D deficiency may contributes to BPPV recurrence. So, some researchers (56, 57) speculated that vitamin D therapy for people with vitamin D insufficiency may lessen the recurrence of BPPV. Rhim (31) et al. established that vitamin D supplementation would lower the recurrence rate throughout 6 months of follow-up, and he concluded that blood vitamin D concentrations significantly impact the recurrence of BPPV. Sheikhzadehi (58) et al. conducted a study demonstrate that oral nutritional therapy with vitamin D3 and antioxidants help decrease relapses in individuals suffering from high recurrence BPPV. The same result was reported in Sánchez et al (27) study, BPPV patients with lower blood vitamin D levels who received vitamin D supplementation showed a substantial decrease in vertigo episodes, which was consistent with our meta-analysis. Our current analysis found that vitamin D supplementation can dramatically minimize recurrence in BPPV patients who with vitamin D insufficiency.

Our meta-analysis showed that vitamin D levels were lower in cupulolithiasis than in canalolithiasis groups, which consistent with Nakada et al. (59) study, which stated the difference in serum vitamin D concentrations between canalolithiasis and cupulolithiasis. These results point to pathophysiological differences between canalolithiasis and cupulolithiasis in relation to vitamin D level. Further investigation is required to uncover the processes behind this phenomena in the future.

In conclusion, our meta-analysis reveals that vitamin D levels are quite important in BPPV patients. Vitamin D may influence calcium homeostasis and bone metabolism, which might impact the creation and function of otoconia in the inner ear. There is no unified theory regarding the mechanism between vitamin D levels and BPPV. The possible mechanisms are as follows: (1) Vitamin D may regulates calcium homeostasis by influencing calcium deposition and dissolution in the inner ear (otoconia) (25). (2) Immunomodulation: All of the body’s cells have the VDR, which plays a role in immunomodulation, cell division, and proliferation (48). It can regulate the function of immune cells, such as T lymphocytes and macrophages. Immune-mediated processes in the inner ear might be involved in the pathophysiology of BPPV. Vitamin D may control the inner ear’s immune response and stop autoimmune responses or excessive inflammation that might harm the vestibular system (48); (3) Antioxidant Activity: In the context of BPPV, oxidative stress could potentially affect the structure and function of otoconia and the vestibular sensory epithelium (60). Vitamin D may protect the inner ear from oxidative stress-induced damage while also maintaining proper vestibular function.

As we know, Vitamin D participates in several physiological processes, includes the regulation of arterial blood pressure, modulation of immune responses, regulation of insulin secretion, protection against certain cancers, renoprotection, and other beneficial effects (61). All elements of the epithelial Ca channel transport system are expressed as transcripts in the cochlea and semicircular canal duct, according to a recent experimental research. Vitamin D increases the otolith of utriculi, despite it being smaller than the bone (62). Similarly, calcium and 25(OH)-D help to assure the integrity of the support tissues’ hair cells. According to some researchers, the onset of recurrent otoconia dislocation may also be related to mechanisms of increased resorption and decreased fixation of calcium. Therefore, a reduction in calcium fixation may lead to deficiencies in the remodeling of the otoconia’s internal structure and their adhesion to the otoconial membrane (63–65). Thus, our research indicates that vitamin D supplementation may lower the recurrence risk of BPPV in individuals with vitamin D insufficiency. In conclusion, since the patients did not suffer from any illnesses that affected their vitamin D intake, conversion, or absorption, we suggest that doctors give BPPV patients the option to check their vitamin D levels and recommend either dietary or medication supplements if necessary.

Despite these findings, the meta-analysis has certain limitations. First, the majority of the papers considered were case–control studies, which are less reliable than randomized controlled trials. Second, because the studies were confined to those published in English, we cannot rule out the possibility of publication bias, despite the funnel plot being mostly symmetrical, indicating no significant danger. Third, future research should look at the potential links between vitamin D levels and BPPV subtypes, season, weather, skin color, lifestyle, nutrition, and vitamin assays. Furthermore, observational studies have reported more substantial effects of vitamin D supplementation than randomized controlled trials. In the future, more RCT (Randomized Controlled Trial) studies will be needed to confirm our conclusions.

## Conclusion

The present meta-analysis evaluates vitamin D levels for BPPV and recurrent BPPV, which indicates that lower vitamin D levels are associated with the incidence and recurrence of BPPV, and vitamin D supplementation in BPPV patients with deficiency or insufficiency reduces recurrence of BPPV. Due to the limited quality and quantity of the listed studies, robust researches with sufficient sample sizes are necessary to validate the result.

## Data Availability

The original contributions presented in the study are included in the article/Supplementary material, further inquiries can be directed to the corresponding author/s.
