# Supplementary material for: Association between vitamin D, vitamin D supplementation and benign paroxysmal positional vertigo: a systematic review and meta-analysis
Source: Front Neurol. 2025 Apr 16;16:1560616. doi: 10.3389/fneur.2025.1560616 (PMC12040629; doi:10.3389/fneur.2025.1560616)
Supplement: Supplementary file 1 [file Data_Sheet_1.PDF]

Association between vitamin D、vitamin D supplementation and benign paroxysmal positional vertigo: a systematic review and meta-analysis

Yanyan Li, Peng Gao, Rui Ding, Xiaorui Pei, Lianhe Li

Citation
Yanyan Li, Peng Gao, Rui Ding, Xiaorui Pei, Lianhe Li. Association between vitamin D、vitamin D supplementation and benign paroxysmal positional vertigo: a systematic review and meta-analysis. Not yet published.

PREVIEW

REVIEW TITLE AND BASIC DETAILS

Review title

Association between vitamin D、vitamin D supplementation and benign paroxysmal positional vertigo: a systematic review and meta-analysis

Condition or domain being studied

Vitamin D; Benign Paroxysmal Positional Vertigo; Vitamin Supplementation

Benign paroxysmal positional vertigo (BPPV) is the predominant kind of peripheral vestibular dysfunction,which has a negative impact on their daily lives.BPPV typically recurs, with a recurrence rate of 15-56% during a 1-10 year period6.This not only results in increasing medical expenses for individuals and their families but also imposes an additional burden on society in terms of healthcare resources utilization. Therefore, it is crucial to ascertain the variables that contribute to the onset and recurrence of benign paroxysmal positional vertigo (BPPV).Numerous investigations have demonstrated that low blood levels of 25-hydroxyvitamin D (25(OH)D) are associated with both the incidence and recurrence of BPPV14-16,vitamin D insufficiency has been extensively studied as a risk factor for the development and recurrence of BPPV. However, the results are inconclusive.The primary purposes of this meta-analysis are to identify the relationship among vitamin D and BPPV occurrence ,vitamin D and BPPV incidence,also summarize the evidence for supplementation of vitamin D on BPPV recurrences.

Rationale for the review

Benign paroxysmal positional vertigo (BPPV) is the predominant kind of peripheral vestibular dysfunction,which has a negative impact on their daily lives.BPPV typically recurs, with a recurrence rate of 15-56% during a 1-10 year period6.This not only results in increasing medical expenses for individuals and their families but also imposes an additional burden on society in terms of healthcare resources utilization. Therefore, it is crucial to ascertain the variables that contribute to the onset and recurrence of benign paroxysmal positional vertigo (BPPV).Numerous investigations have demonstrated that low blood levels of 25-hydroxyvitamin D (25(OH)D) are associated with both the incidence and recurrence of BPPV14-16,vitamin D insufficiency has been extensively studied as a risk factor for the development and recurrence of BPPV. However, the results are inconclusive.The primary purposes of this meta-analysis are to identify the relationship among vitamin D and BPPV occurrence ,vitamin D and BPPV incidence,also summarize the evidence for supplementation of vitamin D on BPPV recurrences.

Review objectives

The primary purposes of this meta-analysis are to identify the relationship among vitamin D and BPPV occurrence ,vitamin D and BPPV incidence,also summarize the evidence for supplementation of vitamin D on BPPV recurrences.

Keywords

Benign Paroxysmal Positional Vertigo BPPV; Vitamin D; Recurrence

Country
China

ELIGIBILITY CRITERIA

Population

Included
Inclusion:The search methodology was confined to publicly accessible data and articles in the English language. Patients who was examined the relationship between vitamin D、vitamin D supplementation and Benign Paroxysmal positional vertigo;

Excluded
Exclusion:non-human subjects

Intervention(s) or exposure(s)

Included
Vitamin D; Nutrient supplementation

Comparator(s) or control(s)

Included
PICO tags selected: Placebo; Vitamin Supplementation

Study design
Only randomized study types will be included.

Context
Studies examined the relationship between vitamin D、vitamin D supplementation and Benign Paroxysmal positional vertigo;non-original research; non-human subjects.

SIMILAR REVIEWS

Check for similar records already in PROSPERO
PROSPERO identified a number of existing PROSPERO records that were similar to this one (last check made on 2 March 2025). These are shown below along with the reasons given by that the review team for the reviews being different and/or proceeding.

- Evaluating the Role of Vitamin D in Preventing Recurrence of Benign Paroxysmal Positional Vertigo: A Systematic Review and Meta-Analysis [published 15 September 2024] [CRD42024587164]. The review was judged **not to be similar**
- Vitamin D supplements for benign paroxysmal positional vertigo [published 14 July 2020] [CRD42020183195]. The review was acknoweldged as **similar** but the authors opted to continue because *the review will be more up to date, the review looks at additional or different outcomes*
- Association between vitamin D deficiency and benign paroxysmal positional vertigo: a systematic review and meta-analysis [published 6 August 2021] [CRD42021271840]. The review was judged **not to be similar**

TIMELINE OF THE REVIEW

Date of first submission to PROSPERO
This record has not been submitted.

Review timeline
Start date: 22 December 2024. End date: 6 February 2025.

Date of registration in PROSPERO

This record has not been published.

AVAILABILITY OF FULL PROTOCOL

Availability of full protocol

A full protocol has been written and uploaded to PROSPERO. The protocol may be accessed through this link https://www.crd.york.ac.uk/PROSPERO/PROSPEROFILES/b0ea52e6a79db75b29d127e566b448ae.pdf.

SEARCHING AND SCREENING

Search for unpublished studies

Only published studies will be sought.

Main bibliographic databases that will be searched

The main databases to be searched are *Embase - Embase via Ovid*, *Embase.com*, *MEDLINE*, *PubMed* and *SCI - Science Citation Index*.

Search language restrictions

The review will only include studies published in English and Chinese.

Search date restrictions

Databases will be searched for articles published before 22 December 2024, there are no restrictions on search start date.

Other methods of identifying studies

Other studies will be identified by: *looking through all the articles that cite the papers included in the review ("snowballing")*, *reference list checking* and *searching conference proceedings*.

Link to search strategy

A full search strategy is available in the full protocol as described in the *Availability of full protocol* section

Selection process

Studies will be screened independently by at least two people (or person/machine combination) with a process to resolve differences.

Other relevant information about searching and screening

None

DATA COLLECTION PROCESS

Data extraction from published articles and reports

Data will be extracted independently by at least two people (or person/machine combination) with a process to resolve differences.

Authors will be asked to provide any required data not available in published reports.

Study risk of bias or quality assessment

Risk of bias will be assessed using: *Newcastle-Ottawa*

A funnel plot was used to determine publication bias.

Data will be assessed independently by at least two people (or person/machine combination) with a process to resolve differences.

Additional information will be sought from study investigators if required information is unclear or unavailable in the study publications/reports.

Reporting bias assessment

Risk of bias due to missing results will be assessed

Certainty assessment

1.Risk of Bias Assessment

A funnel plot was used to determine publication bias.

2.Meta-Analysis and Statistical Methods

Heterogeneity: Whereas heterogeneity across studies was evaluated using Cochrane-based Q and I2 tests. Data followed by p< 0.05 or I 2 > 50% were considered to denote statistically significant heterogeneity, and were subjected to a randomized-effects model. Otherwise, a fixed-effects model was used.

3.Quality assessment:The two reviewers independently assessed the quality and risk of bias of the included studies using the Newcastle-Ottawa score (NOS)

OUTCOMES TO BE ANALYSED

Main outcomes

vitamin D and BPPV occurrence ;

vitamin D and BPPV incidence;

Evidence for supplementation of vitamin D on BPPV recurrences.

Additional outcomes

The level of vitamin D in canalolithiasis than in cupulolithiasis BPPV groups.

PLANNED DATA SYNTHESIS

Strategy for data synthesis

The weighted mean difference (WMD) was used as the metameter and the standard deviation (SD) was considered in evaluating the precision and significance of that point of estimate.Whereas heterogeneity across studies was evaluated using Cochrane-based Q and I2 tests. Data followed by p< 0.05 or I 2 > 50% were considered to denote statistically significant heterogeneity, and were subjected to a randomized-effects model. Otherwise, a fixed-effects model was used. A funnel plot was used to determine publication bias.

CURRENT REVIEW STAGE

Stage of the review at this submission

Review stage	Started	Completed
Pilot work	✓	
Formal searching/study identification	✓	
Screening search results against inclusion criteria	✓	
Data extraction or receipt of IPD		
Risk of bias/quality assessment		
Data synthesis		

Review status

The review is currently planned or ongoing.

Publication of review results

Results of the review will be published in English and Chinese.

REVIEW AFFILIATION, FUNDING AND PEER REVIEW

Review team members

Mrs Yanyan Li (review guarantor and contact) Chaoyang centra hospital. China.

No conflict of interest declared.

Mr Peng Gao. Chaoyang central hospital. China.

No conflict of interest declared.

Mrs Rui Ding. Chaoyang central hospital. China.

No conflict of interest declared.

Mrs Xiaorui Pei. Chaoyang central hospital. China.

No conflict of interest declared.

Professor Lianhe Li. Chaoyang central hospital. China.

No conflict of interest declared.

Named contact

Mrs Yanyan Li (xiaoyanyan19870312@163.com). Chaoyang centra hospital. China.

Review affiliation

Chaoyang central hospital

Funding source

Review has no specific/external funding but is supported by guarantor/review team (non-commercial) institutions.

Peer review

There has been no peer review of this planned review.

ADDITIONAL INFORMATION

Review conflict of interest

Declared individual interests are recorded under team member details.. No additional interests are recorded for this review.

Medical Subject Headings

25-hydroxyvitamin D; Benign Paroxysmal Positional Vertigo; Calcifediol; Dietary Supplements; Humans; Incidence; Risk Factors; Vitamin D; Vitamin D Deficiency; Vitamins

PROSPERO version history

No preview available

Disclaimer

The content of this record displays the information provided by the review team. PROSPERO does not peer review registration records or endorse their content.

PROSPERO accepts and posts the information provided in good faith; responsibility for record content rests with the review team. The guarantor for this record has affirmed that the information provided is truthful and that they understand that deliberate provision of inaccurate information may be construed as scientific misconduct.

PROSPERO does not accept any liability for the content provided in this record or for its use. Readers use the information provided in this record at their own risk.

Any enquiries about the record should be referred to the named review contact
